# RV-PA uncoupling is associated with increased mortality in transthyretin amyloid cardiomyopathy treated with tafamidis

**DOI:** 10.1007/s00392-024-02576-2

**Published:** 2024-11-20

**Authors:** Stéphanie K. Schwarting, Michael Poledniczek, Yuliyan Metodiev, Lukas Stolz, Eva Hofmann, Ute Hegenbart, Stefan Schönland, Stefan Kääb, Steffen Massberg, Norbert Frey, Fabian aus dem Siepen

**Affiliations:** 1 Department of Medicine I, LMU University Hospital Munich, Marchioninistrasse 15, 81377 Munich, Germany; 2 Department of Cardiology, Angiology and Respiratory Medicine, University Hospital of Heidelberg, Im Neuenheimer Feld 410, Heidelberg, Germany; 3 Division of Cardiology, Department of Internal Medicine II, Medical University of Vienna, Vienna, Austria; 4 Department of Internal Medicine V, University Hospital of Heidelberg, Im Neuenheimer Feld 410, Heidelberg, Germany; 5 European Reference Network for Rare, Low Prevalence and Complex Disease of the Heart (ERN GUARD-Heart), Amsterdam, The Netherlands

**Keywords:** RV-PA coupling, Amyloidosis, Transthyretin, Echocardiography, TAPSE/PASP-ratio

## Abstract

**Background:**

The right ventricle to pulmonary artery coupling (RV-PAc) describes the right ventricle’s ability to adjust to an increased afterload. In case of transthyretin amyloid cardiomyopathy (ATTR-CM), impaired RV-PAc can result from increased left ventricular diastolic stiffness due to fibril deposition.

**Objectives:**

While RV-PAc is a validated prognostic parameter in pulmonary arterial hypertension (PAH), its prognostic relevance in ATTR-CM remains unknown. We sought to evaluate the prognostic implications of impaired RV-PAc on survival in ATTR-CM.

**Methods:**

In this multicentre study, RV-PAc was investigated by the echocardiographic surrogate of ratio of tricuspid annular plane systolic excursion to estimated pulmonary arterial systolic pressure (TAPSE/PASP) in 418 ATTR-CM patients, all treated with a TTR stabilizer. The primary outcome was all-cause mortality.

**Results:**

Within a median time of 1.52 [IQR 0.72–2.56] years after diagnosis, 49 (11.7%) patients died. In multivariate Cox regression analysis impaired RV-PAc was a strong independent predictor of mortality (Hazard Ratio (HR) 2.16, 95% Confidence Interval (CI) 1.14–4.07, *p* = 0.018). RV-PAc ratio at first presentation emerged as a robust marker for risk stratification with a determined optimal cut-off of 0.382 mm/mmHg (area under the curve (AUC) 0.73, 95% CI 0.65–0.81). Patients with RV-PAc ratio ≤ 0.382 mm/mmHg exhibited significantly lower survival (HR 4.17, 95% CI 2.21–7.87, *p* < 0.001) within 3 years of follow up than those with RV-PAc ratio above the cut-off (Graphical Abstract).

**Conclusion:**

Impaired adaptation of the RV to increased afterload is associated with worse outcome in ATTR-CM patients. RV-PAc ratio can serve as an echocardiographic predictor for all-cause mortality. Therefore, the determination of RV-PAc could improve risk stratification for ATTR-CM patients.

**Graphical Abstract:**

RV-PA coupling in ATTR-CM patients. This two-center study included patients with transthyretin cardiomyopathy, all treated with specific stabilizer. Tricuspid annular plane systolic excursion (TAPSE, green arrow) to systolic pulmonary artery pressure (PASP, light purple arrows) ratio was investigated retrospectively as correlate for rightventricle to pulmonary artery coupling (RV-PAc). Impaired RV-PAc was associated with higher risk for all-cause mortality, reduced left ventricular function and advanced stages of disease.

**Figure:**
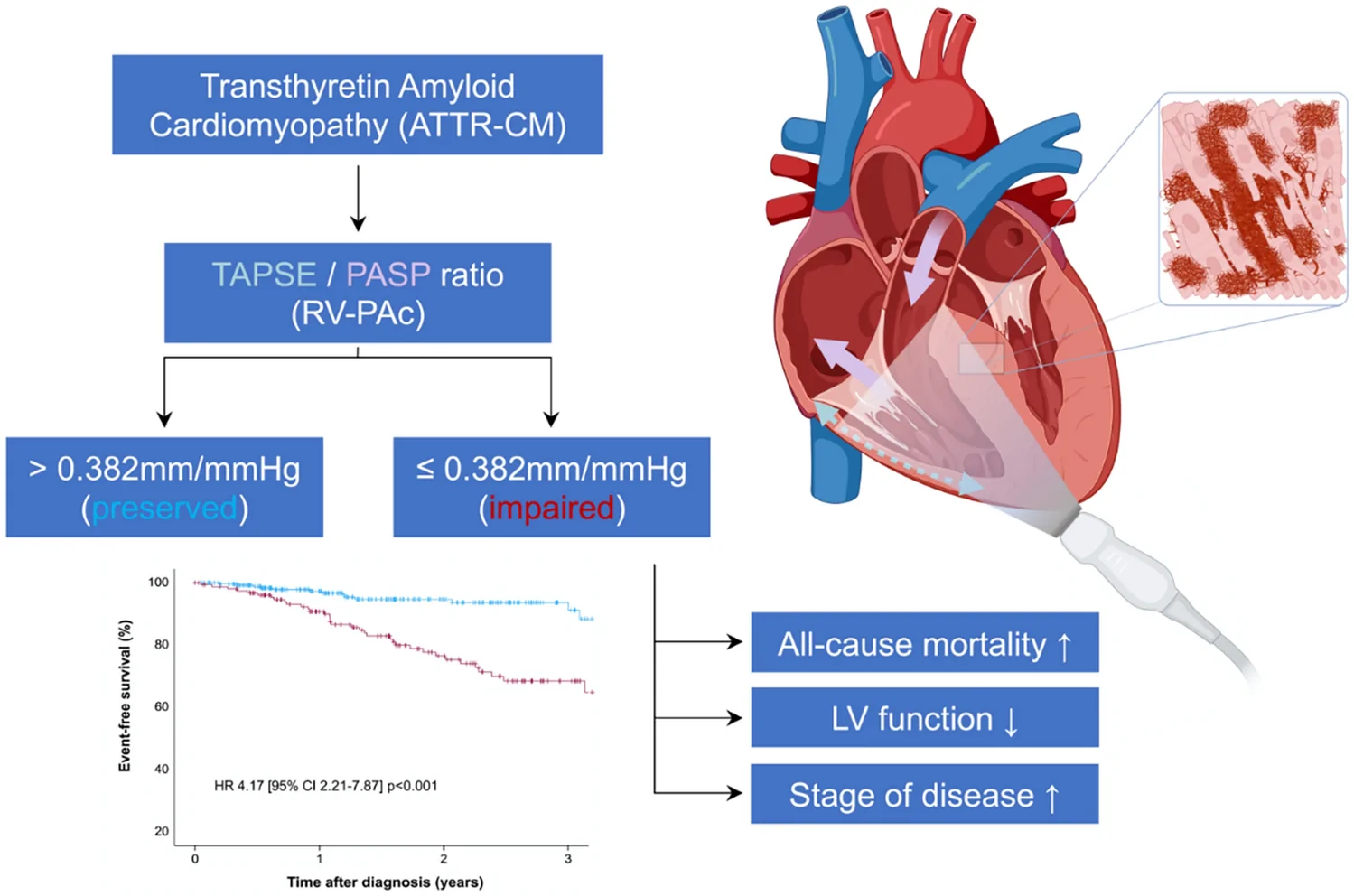

**Supplementary Information:**

The online version contains supplementary material available at https://doi.org/10.1007/s00392-024-02576-2.

## Introduction

Transthyretin amyloid cardiomyopathy (ATTR-CM) is a significantly underdiagnosed cause of heart failure, especially in the elderly [1–3] and can occur as variant type (ATTRv) due to a mutation in the TTR gene or sporadically as wild-type (ATTRwt). Although awareness of ATTR-CM has grown thanks to advances in targeted therapies and the availability of diagnostic tools, understanding of the disease's highly individualized progression remains incomplete. As fibril deposition in heart tissue continues, destruction of the myocardial network and electrical conduction disturbances occur, and patients suffer from recurrent heart failure events. In transthoracic echocardiography, one can observe increased ventricular stiffness and diastolic dysfunction, which transmit elevated pressures in the adjacent cavities such as pulmonary circulation and the right ventricle [4, 5]. The latter has a certain capacity to adapt to increasing afterload, which is presumed to gradually escalate as the disease itself progresses. This ability is referred to as right ventricular to pulmonary artery coupling (RV-PAc) and can readily assessed by the ratio of tricuspid annular plane systolic excursion to estimated pulmonary arterial systolic pressure ratio (TAPSE/PASP) in transthoracic echocardiography [6]. Once hemodynamic stress overdrives the pulmonary circulation, the RV’s capacity becomes depleted, potentially leading to biventricular heart failure. RV-PAc has been extensively investigated in pulmonary arterial hypertension (PAH) [7] and already serves as prognostic marker in pathophysiological conditions such as atrioventricular valve diseases [8–10].

Currently, risk stratification in patients with ATTR-CM is limited to biomarkers, oral diuretic doses and functional assessment [11, 12]. However, the role of imaging, specifically echocardiographic parameters determining the biventricular interplay remain unclear. So, the aim of this study was to assess the mechanisms of right ventricular adaptation in tafamidis-treated ATTR-CM patients and to evaluate its prognostic significance on overall survival.

## Methods

### Study population

This retrospective two-center cohort study included patients with symptomatic ATTR-CM, all presenting between 2020 and 2023. Diagnosis of ATTR-CM was established according to validated criteria in European Society of Cardiology Heart Failure Guidelines [13]. All patients provided written informed consent for participation at local registries and for contact approach to their general practitioner to fulfill the follow up. The patient characteristics, laboratory values, and echocardiographic findings were collected by local investigators at each study site. The censor date was 15 February 2024. The study was approved by the local ethics committees (project numbers 20-0832 and S-091/2022) and the study adheres to the principles outlined in the Declaration of Helsinki.

### Study variables

Demographic data (age and sex), cardiac comorbidities [atrial flutter/fibrillation (Afib), coronary artery disease (CAD)], renal function (estimated glomerular filtration rate), and concomitant medical treatment as well as New York Heart Association (NYHA) functional class were collected. Based on previously published National Amyloidosis Centre (NAC) ATTR Staging system [14], N-terminal pro B-type natriuretic peptide (NT-proBNP) concentration and estimated glomerular filtration rate (eGFR) were used for stratification of the disease. In cases of alternative diuretic medications, a conversion to the equivalent dose of torasemide was performed.

Upon initial presentation, a comprehensive standardized transthoracic echocardiography was performed by experienced investigators using commercially available ultrasound diagnostic systems (GE Healthcare, Milwaukee, WI, USA and EPIQ CVx, Philips Healthcare, Andover, MA, USA). The analysis of the echocardiograms was conducted using locally available software systems compatible with the respective echocardiography machines (Centricity Cardiology CA1000 2.0, GE Medical Systems, Milwaukee, WI, USA and Intellispace Philips Healthcare, Andover, MA, USA) and in line with the respective guidelines [15]. The echocardiographic assessment included LV dimensions as well as functional parameters such as global longitudinal strain (GLS), left ventricular ejection fraction (LVEF), and diastolic dysfunction parameters such as *E*/*A*, *E*/*e′ *lateral and medial at mitral valve annular plane. The right ventricular function was evaluated by tricuspid annular plane systolic excursion (TAPSE). The systolic pulmonary artery pressure (PASP) was estimated by the addition of peak systolic tricuspid regurgitation (TR) gradient with estimated right atrial pressure derived from inferior vena cava width and breath-variability. If tricuspid regurgitation was absent, PASP was assumed being normal with a value of 20 mmHg. The right ventricle to pulmonary artery coupling (RV-PAc) was defined as ratio of TAPSE and PASP. The patients with poor acoustic windows and patients who were not eligible for specific stabilizing therapy were excluded.

### Study endpoints

The primary outcome was all-cause mortality within the first 3 years after diagnosis of ATTR-CM. In a second step, we evaluated symptomatic outcome as expressed by NYHA functional class during follow up.

### Statistical methods

Continuous variables were checked for normal distribution with Shapiro–Wilk-test. Student’s *t*-test was applied for normally distributed, continuous variables; otherwise, Mann–Whitney *U* test was used. Chi-square (χ^2^) test was employed for comparison of categorical variables. Pearson’s *r* was used to assess bivariate correlation between different variables. To identify the optimal cut-off value for prediction of clinical outcomes, we used receiver-operating characteristics (ROC) analysis and Youden’s J. Multivariable cox regression analysis and logistic regression included all parameters presenting with *p* < 0.05 in univariable statistics. Time to combined primary endpoint was depicted in Kaplan–Meier curves. The results are presented as hazard ratio (HR) with 95% confidence interval (CI) and *p*-value. TAPSE and PASP have been excluded from the multivariable regression as both being part of the RV-PA coupling parameter. For statistical analysis, SPSS statistical software (IBM, USA, version 29) was used. Plotting and graphical illustrations were drafted with Affinity Designer (Serif, Europe Ltd, 2019, version 1.10.4).

## Results

### Overall study cohort

**Fig. 1:**
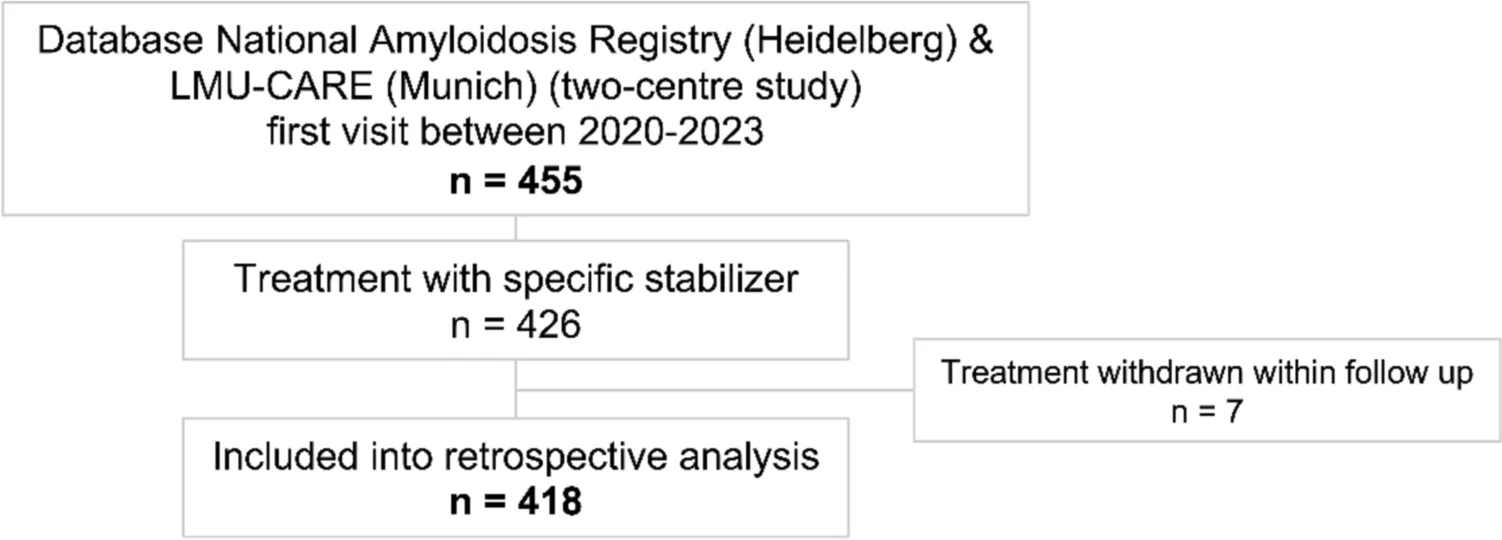
Study flow chart. The two-center study included 418 patients with diagnosis of ATTR-CM and eligible for specific stabilizing therapy. *n* numbers

Among a total of 455 ATTR-CM patients registered at dedicated outpatient cardiac amyloidosis centres between 2020 and 2023, 91.8% (418 patients) were included in this analysis, all treated with specific stabilizing therapy (Fig. 1).

The median age at diagnosis was 79.5 (interquartile range (IQR) [74.5–82.2]) years, with the cohort being predominantly male (91%). The detailed baseline characteristics of the study cohort are outlined in Table 1. The cohort included 22 patients with variant type of ATTR-CM. Most of the patients presented with moderate heart failure symptoms (NYHA functional class I–II in 67%) and only about one fifth (19%, 78 patients) had progressed ATTR-CM according to NAC staging system [14]. In two-thirds of the patient cohorts, concomitant heart failure medication consisted primarily of beta-blockers (ß-blockers) and renin-angiotensin-receptor-antagonists (RAASi). The median diuretic dose was 3.75 [0–5.93] mg per day. The examined cohort exhibited an echocardiographic profile typical for ATTR-CM (Table 2). LV function was mildly reduced as presented by median LVEF of 47 [40–52] % and reduced global longitudinal strain (median GLS -10.6 [8.2–12.9] %). According to median TAPSE (16 [13–20] mm), right ventricular longitudinal function was slightly reduced in the overall cohort.

**Table 1:** Baseline characteristics of the study group

Parameters	Overall (*n* = 418)	RV-PAc preserved TAPSE/PASP ratio > 0.382mm/mmHg (*n* = 260)	RV-PAc impaired TAPSE/PASP ratio ≤ 0.382mm/mmHg (*n* = 158)	*p*-value^a^
Age, y	80 [75–82]	79 [74–82]	80 [77–83]	0.005
Male, *n*	381 (91)	233 (90)	148 (94)	0.157
BMI, kg/m^2^	25.6 [23.5–27.4]	22.4 [20.5–24.4]	22.0 [20.2–23.3]	0.800
ATTRv, *n*	23 (6)	18 (7)	4 (3)	0.052
CAD, *n*	163 (39)	94 (36)	69 (44)	0.127
Afib/flutter, *n*	243 (58)	133 (53)	110 (70)	**< 0.001**
CIED, *n*	54 (13)	28 (11)	27 (17)	
Pacemaker, *n*	47 (11)	25 (6)	23 (15)	
ICD, *n*	7 (2)	3	4	
Heart rate, BPM	73 [64–85]	72 [63–84]	77 [68–85]	**0.001**
≥ 2 AV block, *n*	7 (2)	4	3	
QRS, ms	110 [98–143]	108 [96–141]	117 [100–145]	**0.011**
eGFR, ml/min	60 ± 19	63 ± 19	56 ± 18	**< 0.001**
NT-proBNP, pg/ml	3288 [1449–6172]	1991 [1035–4387]	5552 [3431–8230]	**< 0.001**
NAC stage [14], *n*				**<0.00**
I	180 (43)	147 (57)	31 (20)	**1**
II	160 (38)	79 (30)	82 (52)	
III	78 (19)	34 (13)	45 (28)	
NYHA functional class, *n*				
I	59 (14)	49 (19)	10 (6)	**0.007**
II	221 (53)	139 (54)	82 (52)	
≥ III	138 (33)	72 (27)	64 (42)	
Concomitant HF medication				
ß-blockers, *n*	251 (60)	141 (54)	110 (70)	**0.002**
ACEis/ARBs/ARNIs, *n*	269 (64)	160 (62)	109 (70)	0.124
MRA, *n*	167 (40)	91 (35)	76 (48)	0.008
SGLT-2i, *n*	97 (23)	64 (25)	33 (21)	0.382
Diuretic dose, % of maximum torasemid dose equivalent	3.75 [0–5.93]	5 [0–10]	10 [5–20]	**<0.001**

Values are depicted as mean ± standard deviation, median [IQR] or numbers (%)

*ACEis* angiotensin-converting enzyme inhibitors; *Afib* atrial fibrillation; *ARBs* angiotensin-receptor blockers; *ARNIs* angiotensin-receptor blocker neprilysin-inhibitors; *ATTRv* hereditary transthyretin amyloidosis; *AV block* atrioventricular block; *ß-blockers* betablockers; *BMI* body mass index; *BPM* beats per minute; *CAD* coronary artery disease; *CIED* cardiac implantable electronic device; *eGFR* estimated glomerular filtration rate; *ICD* implantable cardiac defibrillator; *MRA* mineralocorticoid receptor antagonist; *NAC* National Amyloidosis Centre; *NYHA* New York Heart Association; *RV-PAc* right ventricular to pulmonary artery coupling; *SGLT-2i* sodium glucose like transporter 2 inhibitors

^a^*p*-value represents differences between TAPSE/PASP > 0.382 mm/mmHg or ≤ 0.382 mm/mmHg subgroups.

**Table 2:** Echocardiographic characteristics of the study group

Parameters	Overall (*n* = 418)	RV-PAc preserved TAPSE/PASP ratio > 0.382mm/mmHg (*n* = 260)	RV-PAc impaired TAPSE/PASP ratio ≤ 0.382mm/mmHg (*n* = 158)	*p*-value^a^
IVSd, mm	19 [17–20]	18 [16–20]	19 [19–21]	**0.002**
RWT, %	0.74 [0.62–0.87]	0.73 [0.61–0.87]	0.77 [0.66–0.87]	0.098
LVEDD, mm	45 [41–49]	45 [40–49]	45 [41–49]	0.944
LVEF, %	47 [40–52]	49 [42–54]	43 [38–50]	**< 0.001**
LV GLS, –%	10.6 ± 3.2	11.5 ± 3.3	9.4 ± 2.6	**< 0.001**
*E*/*E*′, ms	15 [11–19]	14 [10–18]	17 [13–20]	**< 0.001**
LA diameter, mm	46 [42–50]	45 [41–48]	48 [45–52]	**< 0.001**
MR ≥ 2, *n*	59 (14)	25 (10)	34 (22)	**< 0.001**
RVEDD, mm	38 [34–44]	38 [34–45]	38 [34–43]	0.928
RV free wall thickness, mm	10 [8–12]	10 [8–12]	10 [8–11]	0.511
TR ≥ 3, *n*	10 (2)	4 (2)	6 (4)	0.143
TAPSE, mm	16 [13–20]	19 [15–22]	13 [11–15]	**< 0.001**
PASP, mmHg	35 [24–45]	28 [20–35]	49 [40–59]	**< 0.001**
TAPSE/PASP ratio, mm/mmHg	0.47 [0.31–0.72]	0.61 [0.49–0.90]	0.28 [0.22–0.34]	**< 0.001**

Values are depicted as mean ± standard deviation, median [IQR] or number (%).

*LA* left atrium; *LV GLS* left ventricular global longitudinal strain; *IVSd* end diastolic intraventricular septum diameter; *LVEDD* left ventricular end diastolic diameter; *LVEF* left ventricular ejection fraction; *MR* mitral valve regurgitation; *RVEDD* right ventricular end diastolic diameter; *PASP* systolic pulmonary artery pressure; *RV-PAc* right ventricular to pulmonary artery coupling; *RWT* relative wall thickness; *TAPSE* tricuspid annular plane systolic excursion; *TR* tricuspid valve regurgitation

^a^*p*-value represents differences between TAPSE/PASP > 0.382 mm/mmHg or ≤ 0.382 mm/mmHg subgroups, statistical significance is depicted in bold

### Outcome according to RV-PAc

Within a median time of 1.52 [IQR 0.72–2.56] years, 49 (11.7%) of patients died. The overall survival estimates at 1, 2 and 3 years were 95.5%, 91%, and 89.3%, respectively. The median time between diagnosis and death was 391 [IQR 213–669] days.

**Fig. 2:**
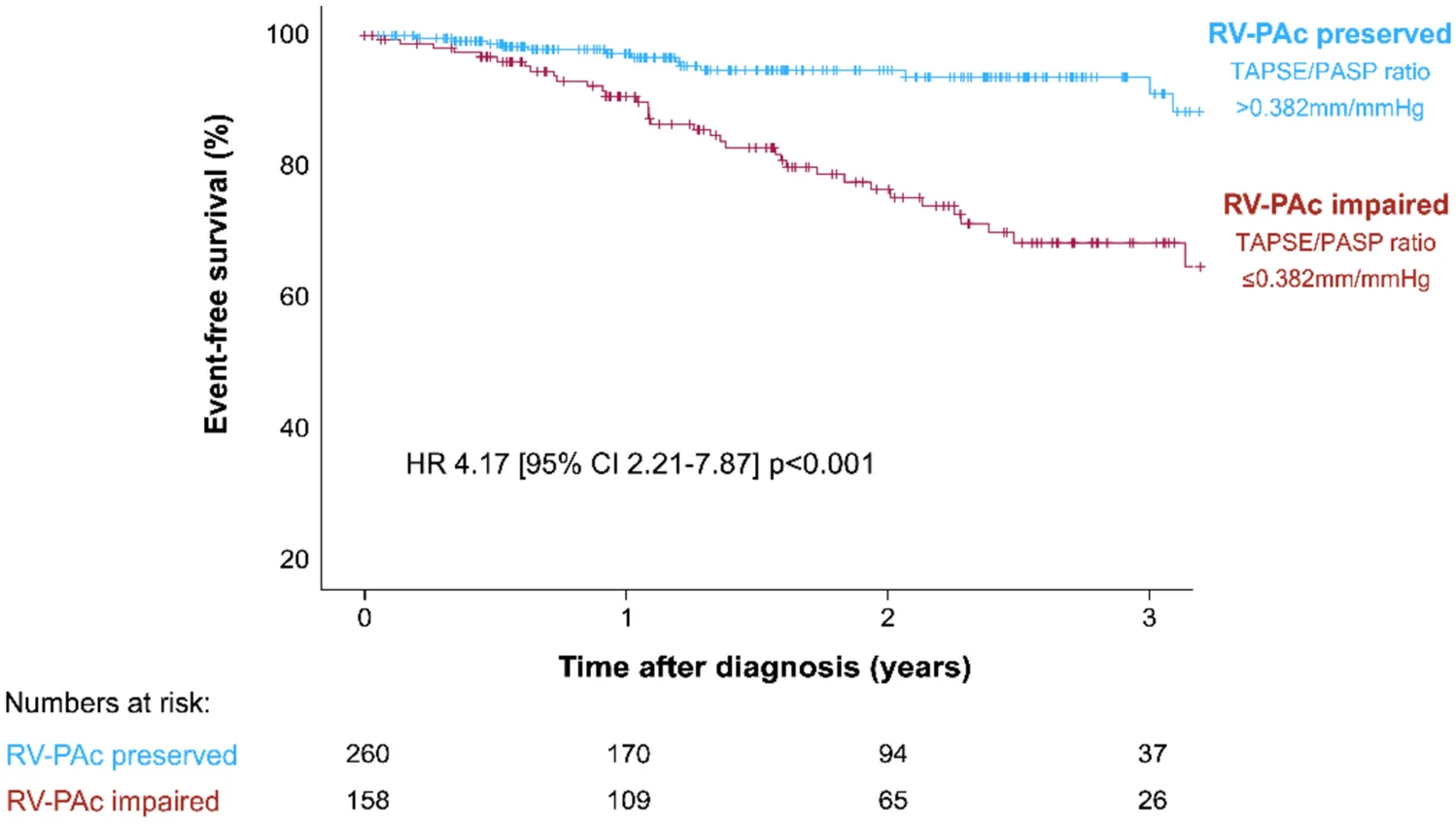
RV-PA uncoupling is associated with worse outcome in ATTR-CM. Survival rate in patients with transthyretin-amyloid cardiomyopathy (ATTR-CM) with impaired right ventricular to pulmonary artery coupling (RV-PAc) (red) than those with preserved RV-PAC (blue). *CI* confidence interval; *HR* hazard ratio; *RV-PAc* right ventricular to pulmonary artery coupling; *TAPSE/PASP* ratio of tricuspid annular plane systolic excursion to estimated pulmonary arterial systolic pressure

In ROC analysis, TAPSE/PASP ratio was a strong discriminator within echocardiographic parameters to predict the primary endpoint, all-cause mortality within 3 years after diagnosis (AUC 0.73, 95% CI 0.65–0.81, Supplemental Figure 1). Youden index was −0.404 (1-specificity 0.7), indicating an optimal RV-PAc cut-off of 0.382 mm/mmHg for discrimination of the primary endpoint. According to this cut-off, the patients were binarily stratified into two groups with RV-PAc > 0.382 mm/mmHg, defined in the following as “preserved RV-PAc” and RV-PAc ≤ 0.382 mm/mmHg, defined as “impaired RV-PAc” (uncoupling), respectively. RV-PA uncoupling was present in 158 patients (38%). Figure 2 shows the Kaplan–Meier chart for overall survival of the two groups within 3 years of follow up.

**Fig. 3:**
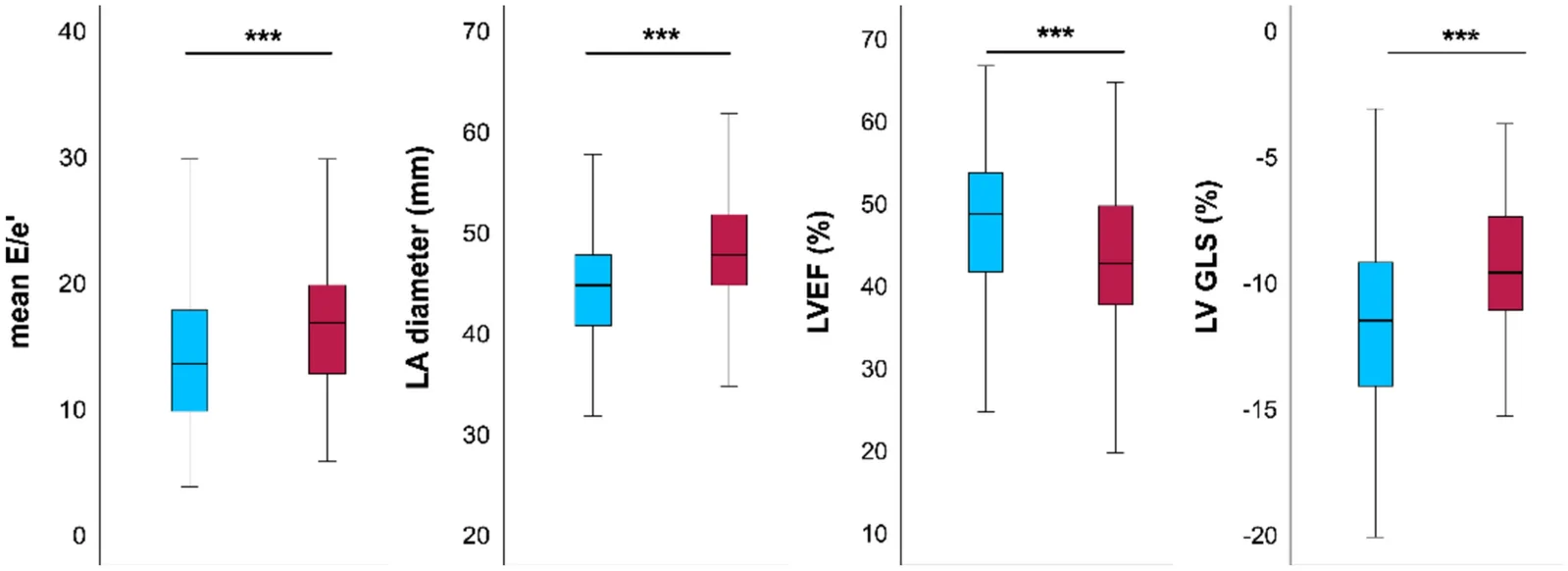
RV-PA uncoupling is associated with impaired left ventricular function in ATTR-CM. ATTR-CM patients with impaired RV-PAc (red) have larger left atria and impaired systolic left ventricle function in comparison to patients with preserved RV-PAc (blue). ***p < 0.001; *LA* left atrium; *LVEF* left ventricle ejection fraction; *LV GLS* left ventricle global longitudinal strain; *mm* millimeter

Patients with impaired RV-PAc (TAPSE/PASP ratio ≤ 0.382 mm/mmHg) had a significantly lower survival (HR 4.17, 95% CI 2.21–7.98, *p* < 0.001) than those with RV-PAc ratio > 0.382 mm/mmHg. The patients with impaired RV-PAc exhibited a significantly advanced stage of the disease, as evidenced by the NAC score (NAC ≥ 2 in 113 (44%) (preserved RV-PAc) vs. 127 (80%) (impaired RV-PAc), *p* < 0.001), significantly higher NYHA functional classes and higher diuretic doses (Table 1). There was a significant association between NAC score and RV-PAc cut-off value (Chi-square (χ2) <0.001). Furthermore, the patients with impaired RV-PAc presented with a prolonged QRS duration, higher heart rate and a higher prevalence of atrial fibrillation at baseline than patients with preserved RV-PAc. In echocardiographic assessment, RV-PA uncoupling was associated with impaired LV function, increased LV wall stiffness according to global longitudinal strain, median E/E’ and left atrium diameter (Fig. 3), whereas there were no significant geometric differences regarding left and right ventricle between both groups (Table 3).

**Table 3:** Multivariate analysis on predictors of overall survival

	Univariate			Multivariate		
	HR	95% CI	*p*-Value	HR	95% CI	*p*-Value
Age, per year	1.10	1.04–1.16	0.001	1.05	0.99–1.12	0.100
NAC stage ≥ 2	7.59	3.01–19.16	<0.001	**3.52**	**1.30–9.56**	**0.014**
Diuretic dose, per mg	1.03	1.02–1.04	<0.001	**1.02**	**1.01–1.03**	**<0.001**
RV-PAc ≤ 0.382 mm/mmHg	4.28	2.39–7.68	<0.001	**2.03**	**1.03–4.02**	**0.042**

Statistically significant results are highlighted in bold.

*Afib* atrial fibrillation; *CI* confidence interval; *HR* hazard ratio; *NAC* National Amyloidosis Centre; *NYHA* New York Heart Association; *RV-PAc* right ventricular to pulmonary artery coupling

Uni- and multivariate Cox regression analysis on established prognostic parameters revealed NAC stage ≥ 2, diuretic dose and RV-PAc ≤ 0.382 mm/mmHg as independent predictors of 3-year all-cause mortality (Table 3). A multivariate Cox regression analysis on echocardiographic parameters (Table 4) revealed LVEF and RV-PAc ≤ 0.382 mm/mmHg as independent echocardiographic parameters to predict outcome within 3 years after diagnosis of ATTR-CM.

**Table 4:** Multivariate analysis on echocardiographic predictors of overall survival

	Univariate			Multivariate		
	HR	95% CI	*p*-Value	HR	95% CI	*p*-Value
LVEF, per %	0.94	0.92–0.97	<0.001	**0.96**	**0.93–0.99**	**0.014**
GLS, per –%	1.21	1.10–1.34	<0.001	1.08	0.96–1.22	0.189
LA diameter, per mm	1.05	1.00–1.10	0.074			
RV-PAc ≤ 0.382 mm/mmHg	4.28	2.39–7.68	<0.001	**3.16**	**1.63–6.12**	**<0.001**

Statistically significant results are highlighted in bold

*CI* confidence interval; *HR* hazard ratio; *LA* left atrium; *LV GLS* left ventricular global longitudinal strain; *LVEF* left ventricular ejection fraction; *MR* mitral valve regurgitation; *RVEDD* right ventricular end diastolic diameter; *RV-PAc* right ventricular to pulmonary artery coupling

### Symptomatic outcome and change in RV-PA coupling

**Fig. 4:**
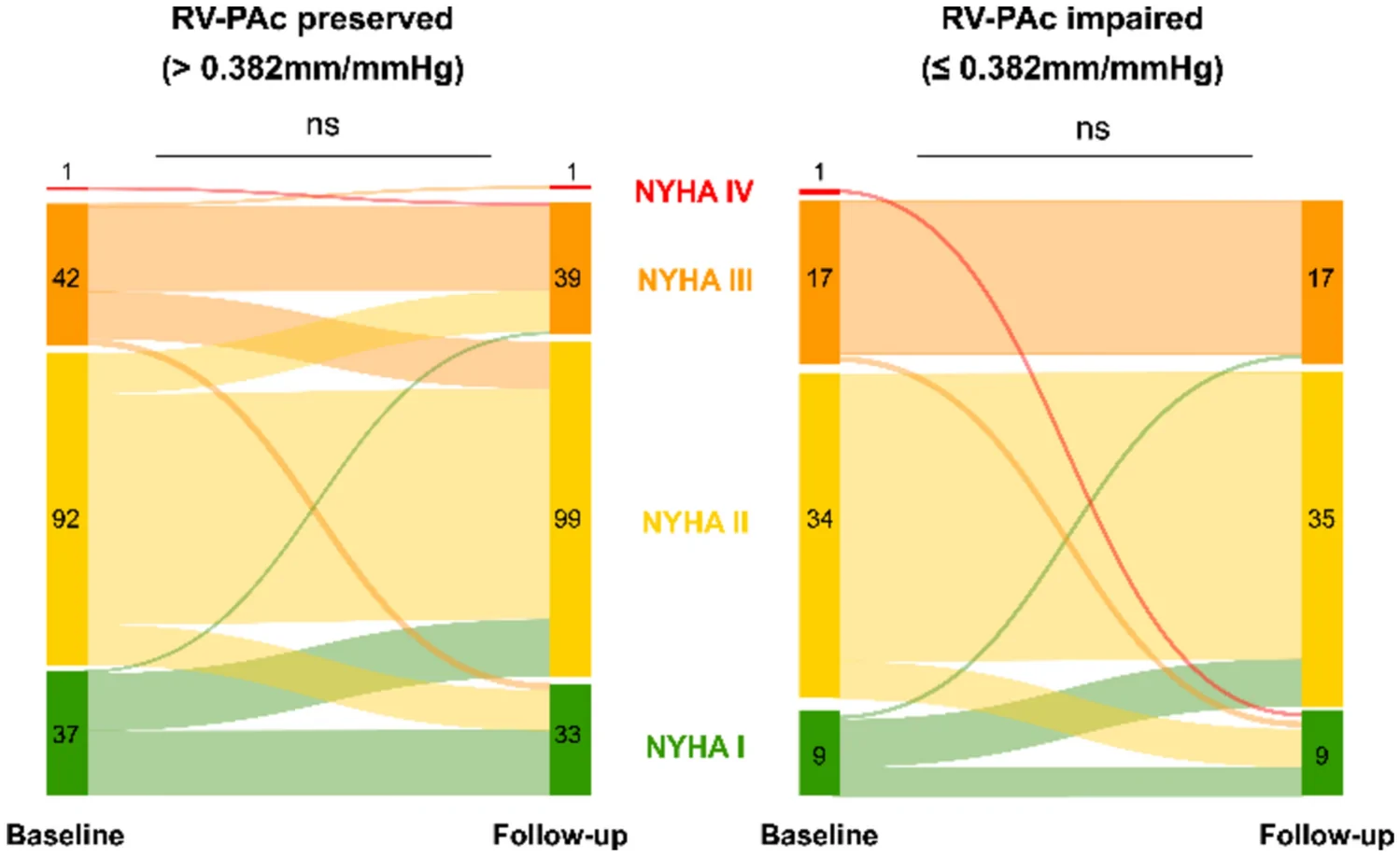
Changes in NYHA functional class according to RV-PAc in ATTR-CM. There was no significant change of New York Heart Association (NYHA) functional class between baseline and follow-up within both groups (preserved (right) and impaired (left) RV-PAc). *ns* not significant; *NYHA* New York Heart Association; *RV-PAc* right ventricle to pulmonary artery coupling

Out of 418 patients, the clinical outcomes based on the NYHA classification could be evaluated in 248 (59%) individuals (*n* = 172 with initially preserved RV-PAc and *n* = 76 with initially impaired RV-PAc) who presented during regular follow-up appointments within the last 6 months before data closure. The median time between baseline assessment and follow up was 594 [373–973] and 583 [399–939] days, respectively. No significant changes in NYHA functional classes were observed according to RV-PAc cut-off value (Fig. 4).

## Discussion

To the best of our knowledge, this is the largest two-centre study to evaluate RV-PAc in the clinical context of ATTR-CM treated with a TTR-stabilizer. In summary, our findings indicate that impaired RV-PAc (cut-off ≤ 0.382 mm/mmHg) (1) is a common feature in advanced stages of the disease and (2) is associated with increased mortality within 3 years following diagnosis of ATTR-CM. This places RV maladaptation in ATTR-CM among the conditions already studied such as atrioventricular valve insufficiencies [16] or aortic stenosis [17, 18], where a left ventricular pathology leads to an increase in resistance within the pulmonary circulation that can significantly worsen patient outcomes.

However, in ATTR-CM, the exact trajectory within the progression of the disease as well as the timing of RV maladaptation onset remains unclear. As ATTR-CM is known to progress gradually, it can be assumed that the RV, although facing increased afterload early on, can also sustain equilibrium over an extended period. Rako et al. [19] and colleagues defined this mechanism as early homeometric adaption which is achieved by concentric hypertrophy with increased RV wall thickness. However, in ATTR-CM it is not possible to differentiate by echocardiographic examination whether RV wall thickening is reactive hypertrophy or pseudohypertrophy upon fibril deposition, currently assumed to be driven by mechano-enzymatic mechanisms such as in increased afterload conditions [20]. Once RV contractility fails to adequately match afterload, homeometric adaption gradually transitions into heterometric adaptation. This process, combined with myocardial stiffening, results in impaired RV function and remodeling induces geometric alterations contributing to ventricular dilation and dysfunction [19]. Interestingly, we did not observe substantial differences in RV geometry between the two groups, suggesting that heterometric adaptation may be somewhat restricted in ATTR-CM and that classical concept of RV contraction patterns [21] and compliance may not fully apply to this specific heart failure etiology.

Previously, Tomasoni and colleagues highlighted the widespread prevalence of bilateral atrioventricular valve regurgitation in ATTR-CM, noting that moderate to severe impairment of these valves is commonly observed in advanced cardiac disease and is associated with poorer outcome [22]. However, our cohort revealed a low prevalence of severe functional tricuspid regurgitation (TR, ≥ grade 3). Consequently, the interplay between RV dysfunction, its compliance, and its pressure–volume characteristics along with the occurrence of TR in ATTR-CM remains poorly understood. This represents a significant unmet medical need, particularly for patients who may undergo interventional edge-to-edge repair of TR.

We identified RV-PAc ≤ 0.382 mm/mmHg as a robust independent echocardiographic predictor of mortality. This cut-off aligns with previous observations on predictive TAPSE/PASP ratios between 0.45 mm/mmHg [23, 24] and 0.31 mm/mmHg [25]. Similar to the findings of other studies, a lower TAPSE/PASP ratio was associated with disease progression itself, depicted by higher NT-proBNP, increased diuretic doses, higher rates of NYHA functional class ≥ 3 and a reduced LV function. However, it is noteworthy that some groups conducted a pooled analysis of light-chain (AL) and TTR amyloidosis. Thus, light chain disease, generally associated with higher risk of mortality, may have biased the ratio during the observation period. Hazard ratio for the composite endpoint of all-cause death or heart failure hospitalization was higher in AL-CA patients (2.47 [1.58–3.85]) than in ATTR-CM (1.81 [1.11–2.95]) [23]. Our study mitigates this influence by exclusively evaluating ATTR-CM patients, all of whom received stabilizer therapy during follow-up.

Based on our analysis, the impaired RV-PA coupling was further associated with elevated heart rates despite higher ß-blocker intake and with significantly prolonged QRS duration. The latter is particularly relevant in the context of delayed LV contraction which hinders right ventricular function by delaying unloading of the pulmonary circulation. Whether resynchronization therapy could lead to an improvement of RV-PA coupling in ATTR-CM has not yet been investigated.

Based on our evaluation of symptoms during follow-up, we demonstrated that despite an impaired RV-PAc, no significant change in NYHA functional class is to be expected within the observation period. Finally, NAC stage and diuretic doses were independent predictors for all-cause mortality next to the RV-PAc cut-off. We can assume that the findings of our study are consistent with real-world data from other larger cohorts and might reflect an additional value of RV-PAc for risk stratification [12].

### Study limitations

The first limitation of this study arises primarily from its retrospective design. Although the investigated cohort offers real-world data, the selection bias occurred as specific treatment was administered only to patients deemed likely to benefit, following criteria outlined in the ATTR-ACT trial [26]. Consequently, the event rate during the 3-year follow-up period was lower compared to previous studies involving patients with ATTR-CM. Second, it should be noted that data on heart failure hospitalizations or cardiovascular mortality are lacking, which are specifically known to occur more frequently in advanced stages of the disease.

Third, while echocardiographic imaging and interpretation were performed by experienced physicians, without adjudication by a core lab, it was assumed that inter-observer variability was low. Fourth, the patients did not undergo right ventricular catheterization (RHC) on regular basis. Thus, correlation with conductance catheter-derived hemodynamic measurements was not feasible. Previous studies on RHC reported that hemodynamic variables are generally elevated in ATTR-CM patients [27], questioning whether classical cut-off values for mean pulmonary artery pressure and right atrial pressure might sufficiently identify patients at higher risk for adverse outcomes. Whether this also features RV-PAc evaluation in transthoracic echocardiography could not be elucidated in our study.

## Conclusion

Our findings identified impaired RV-PA coupling as a relevant prognostic marker for all-cause mortality in ATTR-CM patients treated with stabilizers. The TAPSE/PASP ratio serves as an easily accessible tool to track the course of right ventricular adaptation throughout the disease progression. Given the correlation of RV-PA coupling with progressive disease stages, this parameter has the potential to identify high-risk patients on specific treatments, underscoring the importance of early diagnosis within a clinical framework. Further investigations into RV-PA coupling in ATTR-CM are warranted.

### Clinical perspective

RV-PAc was identified as a novel prognostic marker in ATTR-CM patients treated with a specific stabilizer and is associated with progressive disease stages. It can be easily assessed by tricuspid annulus plane systolic excursion (TAPSE) to estimated systolic pulmonary artery pressure (PASP) ratio and could be included in initial comprehensive transthoracic echocardiographic examination to facilitate risk stratification in ATTR-CM patients. Future studies should assess the feasibility and utility of RV-PAc as a standard prognostic marker in echocardiographic evaluation at the diagnosis of ATTR-CM.

## Supplementary Information

Below is the link to the electronic supplementary material.

Supplementary file1 (DOCX 54 KB)

## Data Availability

Data will be available upon specific request and under specific condition of appropriate collaborative work.
