# Supplementary material for: RV-PA uncoupling is associated with increased mortality in transthyretin amyloid cardiomyopathy treated with tafamidis
Source: Clin Res Cardiol. 2024 Nov 20;115(12):2141–51. doi: 10.1007/s00392-024-02576-2 (PMC13751726; doi:10.1007/s00392-024-02576-2)
Supplement: Supplementary file 1 [file 392_2024_2576_MOESM1_ESM.docx]

**SUPPLEMENTAL MATERIAL**

**Supplemental Figure 1:** **Receiver Operating Curve (ROC) on TAPSE/PASP ratio (RV-PAc).** ROC analysis was performed on TAPSE/PASP ratio for all-cause mortality.

*AUC: area under the curve; CI: confidence interval; RV-PA: right-ventricle to pulmonary artery coupling*
